# Controlled branched-chain amino acids auxotrophy in *Listeria monocytogenes* allows isoleucine to serve as a host signal and virulence effector

**DOI:** 10.1371/journal.pgen.1007283

**Published:** 2018-03-12

**Authors:** Moran Brenner, Lior Lobel, Ilya Borovok, Nadejda Sigal, Anat A. Herskovits

**Affiliations:** Department of Molecular Microbiology and Biotechnology, The George S. Wise Faculty of Life Sciences, Tel Aviv University, Tel Aviv, Israel; The University of Texas Health Science Center at Houston, UNITED STATES

## Abstract

*Listeria monocytogenes* (*Lm*) is a saprophyte and intracellular pathogen. Transition to the pathogenic state relies on sensing of host-derived metabolites, yet it remains unclear how these are recognized and how they mediate virulence gene regulation. We previously found that low availability of isoleucine signals *Lm* to activate the virulent state. This response is dependent on CodY, a global regulator and isoleucine sensor. Isoleucine-bound CodY represses metabolic pathways including branched-chain amino acids (BCAA) biosynthesis, however under BCAA depletion, as occurs during infection, BCAA biosynthesis is upregulated and isoleucine-unbound CodY activates virulence genes. While isoleucine was revealed as an important input signal, it was not identified how internal levels are controlled during infection. Here we show that *Lm* regulates BCAA biosynthesis via CodY and via a riboregulator located upstream to the BCAA biosynthesis genes, named Rli60. *rli60* is transcribed when BCAA levels drop, forming a ribosome-mediated attenuator that *cis*-regulates the downstream genes according to BCAA supply. Notably, we found that Rli60 restricts BCAA production, essentially starving *Lm*, a mechanism that is directly linked to virulence, as it controls the internal isoleucine pool and thereby CodY activity. This controlled BCAA auxotrophy likely evolved to enable isoleucine to serve as a host signal and virulence effector.

## Author summary

Bacterial pathogens must adapt to their host environment to carry out a successful infection. Sensing host-derived signals precedes adaptation, and triggers switching to the virulent state. Within mammalian cells *L*. *monocytogenes* responds to branched-chain amino acids (BCAA) deficiency by inducing virulence gene expression. In this study, we provide compelling evidence that fine tuning BCAA biosynthesis in *L*. *monocytogenes* allows the bacteria to sense isoleucine as a host-specific signal. Tightly controlled BCAA production depends on Rli60, a riboregulator, which is transcribed upstream to the BCAA biosynthesis genes. Rli60 functions as a ribosome mediated attenuator that *cis*-regulates BCAA production under limiting conditions. This study highlights the remarkable cross-regulation of metabolism and virulence in bacterial pathogens.

## Introduction

*Listeria monocytogenes* (*Lm*) is a facultative intracellular bacterial pathogen and the causative agent of listeriosis disease [1]. It invades host cells via phagocytosis, or by induction of endocytosis [2]. Upon invasion, it is initially found in a membrane-bound vacuole, from which it escapes into the host cell cytosol using the pore-forming toxin listeriolysin O (encoded by the *hly* gene) and two phospholipases [3–5]. Once in the host cell cytosol, *Lm* replicates and spreads into neighboring cells using actin-based motility that is mediated by the virulence factor ActA [6,7]. The transcription of the aforementioned virulence factors (and other factors) is regulated by the master virulence activator, PrfA [8].

*Lm* is also a saprophyte, highly abundant in the soil and vegetation. The transition to the pathogenic state relies on sensing of host-specific signals, that together inform the bacterium of its intracellular location. To date, all signals were shown to affect PrfA, directly or indirectly [9]. The signals include temperature [10], availability of carbon sources [11–13], iron [14,15], glutathione [16,17], L-glutamine [18] and BCAA (isoleucine, leucine and valine) [19,20]. We previously found that BCAA, particularly isoleucine, are important metabolic signals for *Lm* in the mammalian niche. *Lm* senses the low availability of BCAA within the host cell cytosol and responds by triggering virulence gene expression [19]. This response is dependent on the global transcription regulator and metabolic sensor, CodY, which directly binds isoleucine and activates or represses genes [21,22]. While classically CodY was shown to gain function upon binding of isoleucine, acting as repressor of metabolic genes, we found it retains a regulatory activity also when unbound to isoleucine [23]. Under this condition, CodY repression of the metabolic genes is alleviated and the unbound CodY becomes an activator of PrfA and thereby the downstream virulence genes [19,20,23]. Notably, while these findings placed CodY at the crossroad of metabolism and virulence, they revealed isoleucine to be a key signaling molecule within the host that influences gene expression. This discovery prompted us to hypothesize that BCAA biosynthesis in *Lm* must be tightly regulated. BCAA biosynthesis in *Lm* is strongly repressed by CodY under high BCAA conditions, and is transcriptionally up-regulated when BCAA levels drop [19,23]. Notwithstanding, despite encoding all the BCAA biosynthesis genes, *Lm* still requires BCAA supplement to support optimal growth under nutrient limiting conditions [24,25]. Considering this observation, we speculated that *Lm* may have evolved additional mechanisms that finely tunes BCAA biosynthesis, enabling isoleucine to serve as a host signal and effector of virulence.

**Fig 1:**
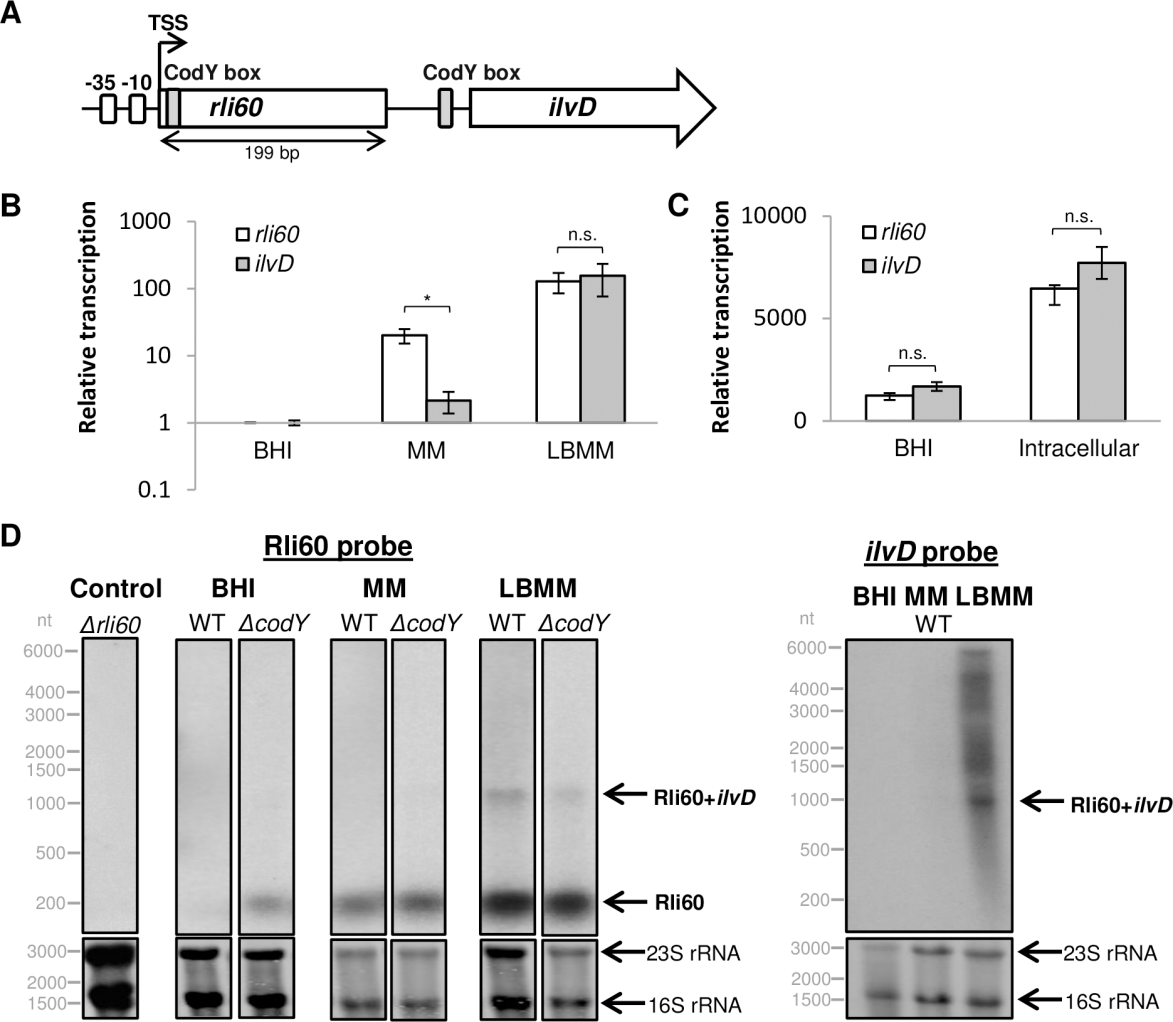
*rli60* and *ilvD* transcription pattern under varying BCAA concentrations. **(A)** Schematic representation of *rli60* and *ilvD (LMRG_01131*) genomic region. A single promoter is indicated upstream to *rli60* (-10 and -35 are marked as white boxes). Two putative CodY binding sites are indicated upstream to *rli60* and to *ilvD* (grey boxes). The transcription start site (TSS), based on a 5’-RACE analysis (see S1 and S2 Figs), is marked with an arrow. **(B)** Comparative qRT-PCR analysis of *rli60* and *ilvD* mRNA levels in WT bacteria grown in BHI, MM and LBMM media. *rli60* and *ilvD* mRNA levels were normalized to the levels of *rpoD* mRNA and to the transcription level in BHI. The data represent 3 biological replicates (N = 3). Error bars indicate standard deviation. represent *P*-values (* = P<0.05, n.s. = non-significant), calculated using Student’s *t*-test. **(C)** Transcription analysis of *rli60* and *ilvD* mRNA in WT bacteria grown in BHI and in bone marrow derived macrophages (BMDM) using a Nanostring analysis. The data represent 2 biological replicates (N = 2). Error bars indicate standard deviation. *P*-values (n.s. = non-significant) were calculated using Student’s *t*-test. **(D)** Northern blot analysis of *rli60* and *ilvD* mRNA transcripts using ^32^P-labeled specific probes. Total RNA was extracted from WT *Lm* and *ΔcodY* bacteria grown in BHI, MM and LBMM and hybridized with Rli60 (left panel) or *ilvD* (right panel) probe. 23S and 16S rRNA were used as a loading control, and *Δrli60* RNA extracts as a negative control. All samples were tested on the same membrane, and were separated only for visualization.

Several transcriptome studies identified a putative small RNA, named Rli60, upstream to the BCAA biosynthesis genes (Fig 1A) [23,26–28]. Rli60 was predicted to function as a riboswitch [26] or as a sRNA [27], though these predictions were not validated. Other studies suggested a role for Rli60 in biofilm formation and virulence, by a mechanism that is not known [29,30]. Here we found that Rli60 functions as a ribosome-mediated attenuator that *cis*- regulates BCAA biosynthesis genes. Importantly, we found this riboregulator to restrict BCAA production even under BCAA depletion. This property is important for *Lm* virulence, as it limits the internal pools of BCAA, thus maintaining isoleucine signaling function via CodY. This controlled BCAA-auxotrophy in *Lm* may thus represent an adaptive mechanism to the life within the host.

## Results

### *rli60* is co-transcribed with *ilv-leu* genes under low BCAA conditions

In *Lm* the BCAA biosynthesis genes are encoded in one operon consisting of nine genes (*ilvDBHC-leuABCD-ilvA*), named the *ilv-leu* operon. *rli60* transcript was previously identified upstream to *ilvD*, the first gene of this operon, and was suggested to consist of 184 to 339 nt, raising the question whether it functions as a sRNA or *cis*-regulatory element [26–28]. Examining the *rli60-ilvD* genomic region, we identified a single promoter upstream to *rli60* with no additional promoter upstream to *ilvD* (Fig 1A and S1 Fig). Rapid amplification of cDNA 5'-Ends (5'-RACE) analysis confirmed that *rli60* and *ilvD* are co-transcribed and share a single transcription start site (TSS) (Fig 1A and S1 and S2 Figs). Importantly, the co-transcript was detected under BCAA limiting conditions, whereas a shorter transcript (~200 nt) representing only Rli60 RNA was detected under rich BCAA conditions, suggesting a BCAA-dependent transcription regulation (S2 Fig).

We employed quantitative reverse-transcription PCR (qRT-PCR) to analyze the transcription pattern of *rli60* and *ilvD* in *Lm* bacteria grown under varying BCAA concentrations. Three types of media were used: brain heart infusion (**BHI**), a rich medium containing excess amounts of BCAA; a minimal defined medium (**MM**) containing 800 μM of each BCAA; and a low BCAA minimal defined medium (**LBMM**) containing 80 μM of each BCAA. As shown in Fig 1B, under rich BCAA conditions (*i*.*e*., in BHI) both *rli60* and *ilvD* were repressed, whereas under low BCAA conditions (*i*.*e*., in LBMM) their transcription was up-regulated (~140-fold). Notably, in the MM medium a differential transcription pattern was observed, where *rli60* exhibited a higher transcription level in comparison to *ilvD*, suggesting a premature termination of transcription may occurs upstream to *ilvD*. Further analysis of *rli60*-*ilvD* transcription in bacteria grown intracellularly in bone marrow-derived macrophage cells (BMDM) revealed a similar transcription pattern to that seen upon *Lm* growth in LBMM (Fig 1C), insinuating low availability of BCAA within the macrophage cytosol. Of note, *ilvD* up-regulation was specific to conditions were BCAA were limited, and was not observed upon limitation of other amino acids *e*.*g*., arginine, or both tryptophan and phenylalanine [19] (S3 Fig).

To further corroborate the premise that *rli60* and *ilvD* are regulated in a BCAA-dependent manner, Northern blot analyses of Rli60 and *ilvD* were performed on RNA extracted from WT bacteria grown in BHI, MM and LBMM. This analysis confirmed the existence of Rli60 RNA at the size of ~200 nt, and its transcription under BCAA limiting conditions (Fig 1D). In accordance with the 5’-RACE and the qRT-PCR analyses, a longer transcript of a ~1000 nt was detected in LBMM (Fig 1D). Northern blot analysis using an *ilvD* specific probe suggested that this ~1000 nt transcript may represent the *rli60-ilvD* co-transcript. Additional longer transcripts of the *ilv-leu* operon were also detected, not including *rli60*, suggesting it may be processed (cleaved). (Fig 1D). Altogether, these findings establish that *rli60* is co-transcribed with the *ilv-leu* genes in a BCAA-dependent manner, suggesting it may function as a *cis-*regulatory element. To address the question whether *rli60* is also repressed by CodY under high BCAA conditions, as known for the *ilv-leu* genes [19], a Northern blot analysis of Rli60 was performed on RNA extracts from Δ*codY* bacteria. Higher levels of Rli60 were observed in Δ*codY* bacteria under high BCAA conditions compared to WT bacteria, demonstrating that CodY represses *rli60* when BCAA are plentiful (Fig 1D). Accordingly, two putative CodY binding-sites were identified upstream to *rli60* and *ilvD* genes (Fig 1A and S1 Fig).

### Both Rli60 and CodY negatively regulate BCAA biosynthesis

**Fig 2:**
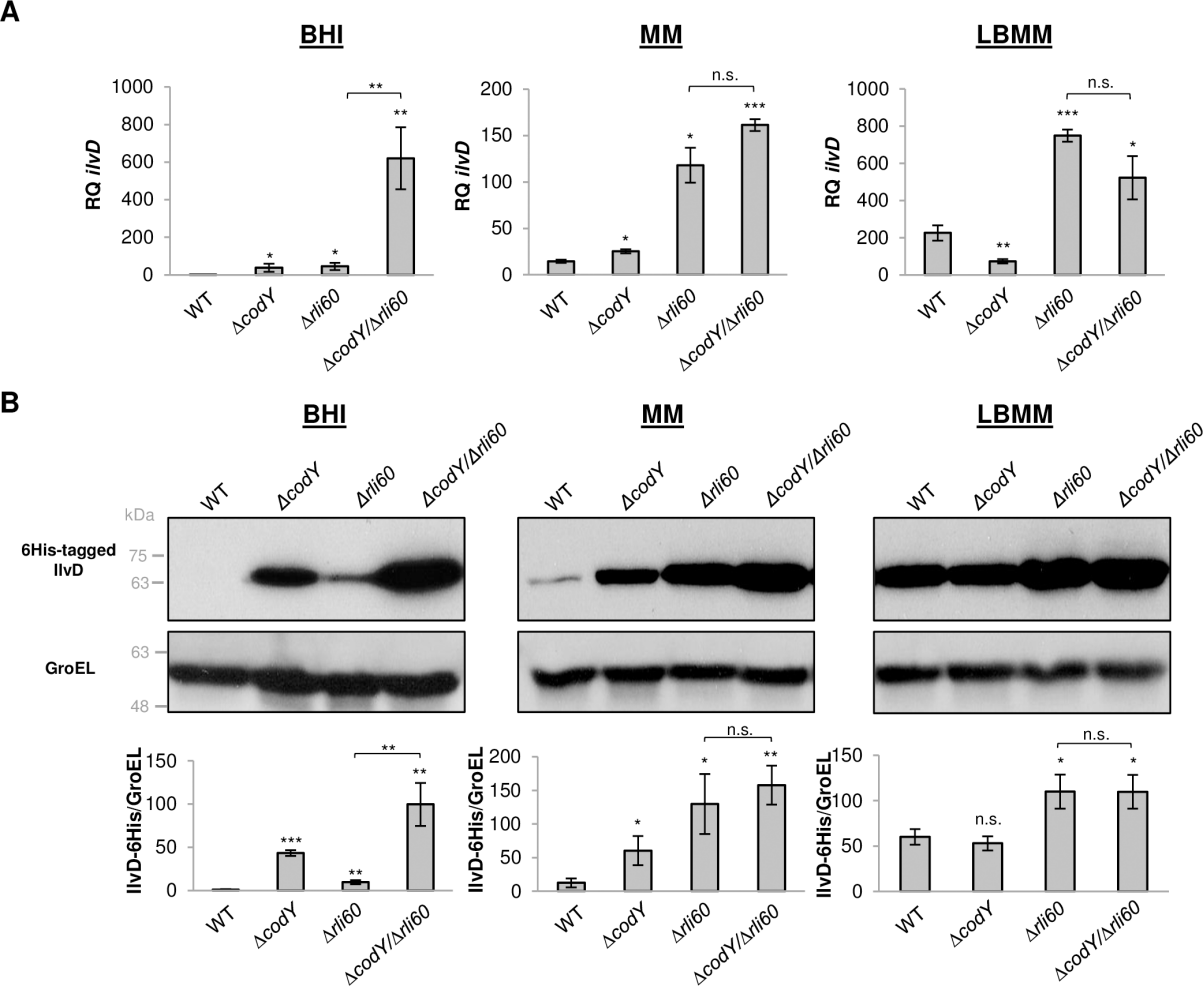
Both Rli60 and CodY negatively regulate the BCAA biosynthesis pathway. **(A)** qRT-PCR analysis of *ilvD* transcription level in WT *Lm*, *ΔcodY*, *Δrli60* and *ΔcodY/Δrli60* bacteria grown in BHI, MM and LBMM. *ilvD* mRNA levels were normalized to *rpoD* mRNA and are represented as relative quantity (RQ), relative to *ilvD* mRNA level in WT bacteria grown in BHI. The data represent 3 biological replicates (N = 3). Error bars indicate standard deviation. Asterisks represent *P*-values (* = P<0.05, ** = P<0.01, *** = P<0.001, n.s. = non-significant), calculated using Student’s *t*-test. P-values represent a comparison to the respective WT sample, unless indicated otherwise. **(B)** Western blot analysis of 6His-tagged IlvD protein in WT *Lm*, *ΔcodY*, *Δrli60* and *ΔcodY/Δrli60* bacteria grown in BHI, MM and LBMM media. Anti-His antibody was used to probe the 6His-tagged IlvD. Detection of GroEL was used as a loading control. A representative blot is presented in the upper panel and densitometry analyses of 3 independent experiments are shown in the lower panel. IlvD-6His protein levels are relative to protein level in WT bacteria grown in BHI. Protein levels were normalized to GroEL protein. The data represent 3 biological replicates (N = 3). Error bars indicate standard deviation. Asterisks represent P-values (* = P<0.05, ** = P<0.01, *** = P<0.001, n.s. = non-significant) calculated by Student's *t*-test. P-values represent a comparison to the respective WT sample, unless indicated otherwise.

To further characterize the role of Rli60 as a regulator of the *ilv-leu* operon and its relationship with CodY, we examined the transcription of *ilvD* in WT, Δ*codY*, Δ*rli60* and in a Δ*codY*/Δ*rli60* double mutant strain under the different BCAA growth conditions. Under high BCAA conditions (*i*.*e*., in BHI), Δ*codY* and Δ*rli60* mutants transcribed *ilvD* to a similar level (~40-fold more than WT bacteria), whereas the double mutant (Δ*codY*/Δ*rli60*) up-regulated *ilvD* transcription by ~600-fold, as compared to WT bacteria (Fig 2A). In MM medium, where the BCAA levels are lower, *ilvD* was only slightly upregulated in Δ*codY* in comparison to WT bacteria, since under this condition CodY repression is lessened (Fig 2A). Remarkably, Rli60 was found to be the main repressor of the *ilv-leu* genes under this condition, as evidenced by the enhanced *ilvD* transcription in Δ*rli60* and Δ*codY*/Δ*rli60* bacteria (~10-fold in comparison to WT bacteria) (Fig 2A). Upon low BCAA conditions (*i*.*e*., in LBMM), *ilvD* transcription was upregulated in WT bacteria (~220-fold as compared to WT bacteria grown in BHI) (Fig 2A), since under this condition the transcription continues through *rli60*, transcribing the *ilv-leu* genes (Fig 1B and 1D). That said, Rli60 still repressed the *ilv-leu* genes under this condition, as *ilvD* transcription was even higher in Δ*rli60* and Δ*codY*/Δ*rli60* bacteria (by ~3-fold), overall indicating that Rli60 prevents the full activation of this operon (Fig 2A). Taken together, these findings suggest that two BCAA-dependent mechanisms regulate the *ilv-leu* operon; the first is CodY, which represses both *rli60* and the *ilv-leu* genes under high BCAA conditions, and the second is Rli60, which kicks in when BCAA levels drop, further repressing the transcription of the *ilv-leu* genes. Notably, an overall similar expression pattern was observed with IlvD protein, under the same growth conditions and strains, using Western blot analysis (Fig 2B), corroborating the premise that CodY and Rli60 are the primary regulators of BCAA biosynthesis.

### Rli60 functions as a ribosome-mediated transcription attenuator

**Fig 3:**
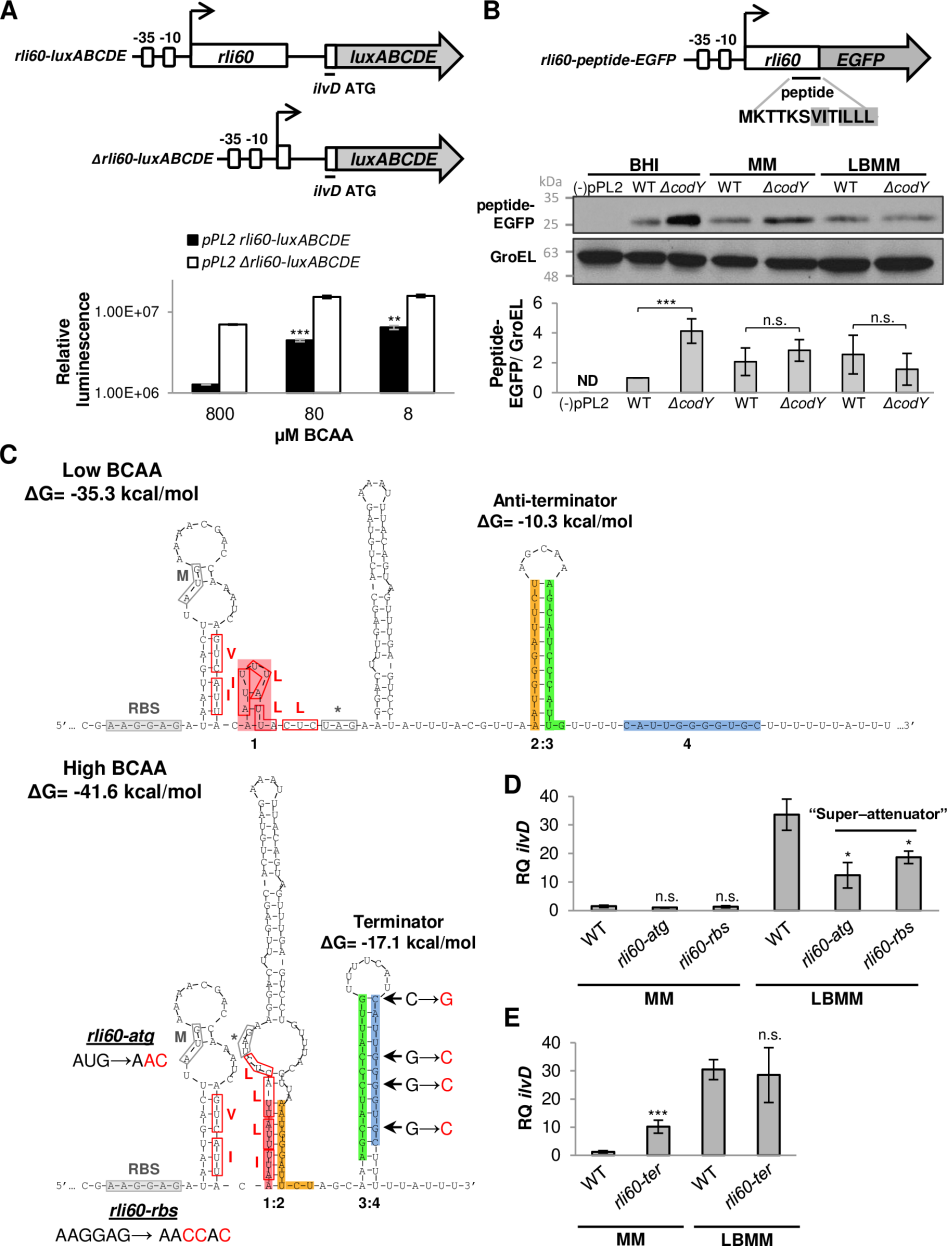
Rli60 functions as a ribosome-mediated transcriptional attenuator. **(A)** The regulatory region of *ilvD* (675 bp upstream to *ilvD* start codon) harboring *rli60* or deleted of *rli60* was fused to a luciferase reporter system (*luxABCDE*). Schematic representation of the two reporter systems are shown. On the lower panel; luminescence measurements of *E*. *coli* bacteria auxotrophic for BCAA (K-12 *ilvC*::*Km*), harboring pPL2*-rli60-luxABCDE* or pPL2*-Δrli60-luxABCDE* plasmid, grown in MM medium supplemented with 8, 80, or 800 μM of each BCAA. Results are presented as relative luminescence units normalized to the bacterial relative growth (OD_600_ values). The Y-axis is presented at logarithmic scale. The data is representative of 3 biological replicates (N = 3). Error bars represent standard error of a technical duplicate. Asterisks represent *P*-values (* = P<0.05, ** = P<0.01, *** = P<0.001, n.s. = non-significant), calculated using Student’s *t*-test. P-values represent a comparison to the 800 μM sample, unless indicated otherwise. **(B)** A translational fusion of *rli60*-leader peptide (13 aa) to enhanced green fluorescent protein (EGFP). A schematic representation of the reporter system is shown. A Western blot analysis of EGFP-leader peptide in WT and *ΔcodY* bacteria, harboring pPL2-*rli60*-peptide-EGFP, grown under the different media. (-)pPL2 denotes bacteria that were not conjugated with the plasmid as a negative control. Anti-GFP antibody was used for the detection of EGFP protein, and detection of GroEL was used as a loading control. A representative blot is presented. Densitometry analyses of 3 independent experiments are shown. Peptide-EGFP protein levels were normalized to GroEL protein and to the respective WT BHI sample (ND = not detected). The data represent 3 biological replicates (N = 3). Error bars indicate standard deviation. Asterisks represent *P*-values (*P<0.05, **P<0.01, ***P<0.001, n.s. = non-significant), calculated using Student’s *t*-test. **(C)** Identification of 2 alternative RNA structures in Rli60 using PASIFIC [32]. Four segments are represented in colors demonstrating the overlapping hairpins that are formed (segment 1-pink, 2-orange, 3-green and 4-blue). The first structure in the upper panel stabilizes an antiterminator hairpin (segments 2 and 3) exhibiting a ΔG of -10.3 kcal/mol. The second structure in the lower panel stabilizes a terminator haipin (segments 3 and 4) exhibiting a ΔG of -17.1 kcal/mol. The ribosome binding site (RBS), start codon, stop codon and the regulatory codons of the leader peptide are marked in gray and red boxes, in both structures. The stop codon is indicated by an asterisk. Point mutations are marked in red next to each feature. **(D)** RT-qPCR analysis of *ilvD* transcription level in WT, *rli60-atg* and *rli60-rbs* bacteria grown in MM and LBMM media. *ilvD* mRNA levels were normalized to *rpoD* mRNA levels and are represented as relative quantity (RQ), relative to the respective WT sample. The data represent 3 biological replicates (N = 3). **(E)** RT-qPCR analysis of *ilvD* transcription level in WT and *rli60-ter* bacteria grown in MM and LBMM media. *ilvD* mRNA levels were normalized to *rpoD* mRNA levels and are represented as relative quantity (RQ), relative to the respective WT sample. The data represent 3 biological replicates (N = 3). Error bars represent standard deviation. Asterisks represent *P*-values (* = P<0.05, ** = P<0.01, *** = P<0.001, n.s. = non-significant), calculated using Student’s *t*-test. *P*-values represent a comparison to the respective WT sample.

We reasoned that Rli60 may have the ability to directly sense BCAA and to act as a *cis-*regulatory RNA. To investigate this hypothesis, *rli60* in the context of its native regulatory region (consisting 675 bp upstream to IlvD start codon) was cloned upstream to luciferase reporter genes on a pPL2 plasmid (pPL2-*rli60-luxABCDE*), which was further transformed into *E*. *coli* bacteria auxotrophic for BCAA (*E*. *coli* K-12 *ilvC*::*Km* strain) (Fig 3A). Using this heterologous system, Rli60 regulation of the downstream *lux* genes could be examined under varying BCAA concentrations (supplemented in the media) in the absence of *de novo* BCAA synthesis and CodY (*E*. *coli* bacteria are devoid of CodY, as it is a Gram-positive specific regulator). We observed that luminescence increased as BCAA concentrations were lowered (Fig 3A), demonstrating that Rli60 directly senses BCAA availability and regulates its downstream genes in a concentration-dependent manner. As a control, a similar plasmid was used, this time deleted of *rli60* sequence (pPL2-Δ*rli60-luxABCDE*), which demonstrated high luminescence levels independent of BCAA concentrations (Fig 3A). These findings indicated that Rli60 directly senses BCAA levels and accordingly negatively regulates its downstream genes.

Taken together, the data suggested that Rli60 *cis-*regulates BCAA biosynthesis in response to BCAA availability, but the mode of regulation was not clear. To search for clues for the regulation type, we examined *rli60* sequence and found a short coding sequence of 13 amino acids that is followed by putative stem-loop structures. Notably, the identified peptide was enriched in BCAA codons (Fig 3B), implying that a reduced rate of translation caused by BCAA limitation may lead to a transcription attenuation. In such a mechanism of ribosome-mediated attenuation, the translation rate of the leader peptide dictates the secondary structure of the leader transcript. When the regulatory amino acids are in short supply, translation is slow, allowing the RNA to form an anti-terminator structure that permits transcription to continue into downstream genes; however, when amino acids supply is in excess, translation is rapid, preventing the formation of the anti-termination loop and causing the RNA to assume a terminator structure [31].

To examine the existence of the leader peptide in *rli60*, it was fused to EGFP (a translational fusion) with its native promoter and cloned into pPL2 plasmid (pPL2-*rli60-peptide-EGFP*) (Fig 3B). Translation of the fused protein was analyzed in WT and Δ*codY* bacteria grown in BHI, MM, and LBMM using Western blot analysis. We observed that the fused protein was indeed translated and that its expression is CodY-dependent under BHI (high BCAA conditions), similarly to Rli60 (Fig 3B). The fused protein was also detected under MM and LBMM conditions, but as expected, to a lower extent. Next, we analyzed Rli60 sequence using PASIFIC server [32] and identified two alternative RNA structures downstream to the leader peptide, consisting of overlapping hairpins that may serve as a terminator and an anti-terminator (Fig 3C). We then performed a mutational analysis of the hairpins in the *Lm* genome. A series of nucleotide substitution mutations were made in the putative terminator hairpin to impair its stability, that do not interfere with the anti-terminator structure (*rli60-ter* mutant) (Fig 3C). Additional mutations were made in the peptide’s ribosome binding site (RBS) and start codon (ATG) to hinder its translation (*rli60-rbs* and *rli60-atg* mutants, respectively) (Fig 3C). The latter mutations are known as “super-attenuators”, as in the absence of engaging ribosomes the terminator hairpin is hyper-stabilized, leading to a premature termination [33]. We next used these mutants to analyze *ilvD* transcription during growth in MM and LBMM, conditions in which *rli60* is transcribed (Fig 1B and 1D). As predicted, under LBMM conditions (where the anti-terminator should be formed) the "super-attenuator" mutants (*rli60-atg* and *rli60-rbs*) demonstrated a significant reduction in *ilvD* transcription (Fig 3D), whereas under MM conditions (where the terminator should be stabilized) the *rli60-ter* mutant demonstrated an enhanced *ilvD* transcription (Fig 3E). As expected, no significant effect was observed for each mutant in the other medium (Fig 3D and 3E), suggesting Rli60 regulates the *ilv-leu* operon via a ribosome-mediated attenuation mechanism.

### Rli60 is the cause for *Lm* BCAA auxotrophy

**Fig 4:**
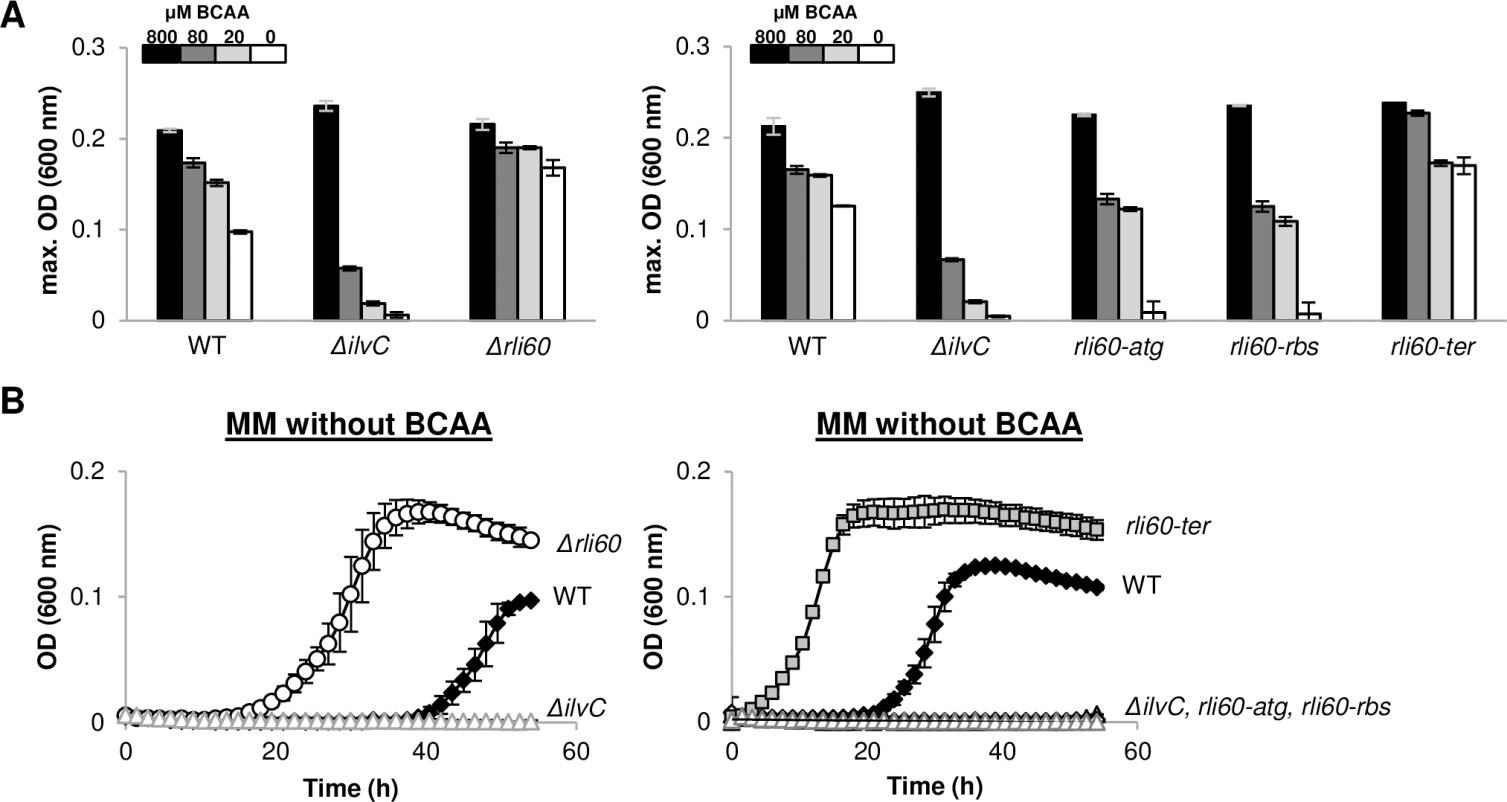
Rli60 mediates *Lm’s* BCAA partial-auxotrophy. **(A)** Presentation of maximal OD measurements (600 nm) of WT bacteria and indicated mutants grown in minimal defined medium containing decreasing concentrations of BCAA (800 μM, 80 μM, 20 μM and without a supplement of BCAA). Maximal OD values were extracted from growth analyses presented in Fig 4B and S4 Fig. The data represent 3 biological replicates (N = 3). Error bars represent standard deviation. **(B)** Growth of WT *Lm* and indicated mutants in minimal defined medium, which is completely lack of BCAA. Growth was measured by a Synergy HT BioTek plate reader at 37°C for 55 h. The data represent 3 biological replicates (N = 3). Error bars represent standard deviation.

In the literature, *Lm* is described as a BCAA auxotroph, or a partial auxotroph, since it requires BCAA supplement for optimal growth [25]. To examine whether Rli60 is the cause for BCAA requirement in *Lm*, we compared the growth of WT, *Δrli60* and *ΔilvC* bacteria in minimal medium supplemented with increasing concentrations of BCAA (0, 20, 80 and 800 μM of each) (Fig 4A and S4 Fig). Of note, *ilvC* encodes a central enzyme in the BCAA biosynthesis pathway [19]. We found that *ΔilvC* behaves like a true auxotroph, failing to grow when BCAA levels drop, whereas WT bacteria exhibit a moderate phenotype, behaving like semi-auxotrophs, and *Δrli60* bacteria grow like prototrophs, less affected by external BCAA levels (Fig 4A and S4 Fig). The different phenotypes were most evident under conditions where no BCAA were added, as *Δrli60* exhibited a significant growth advantage over WT bacteria, while *ΔilvC* did not grow (Fig 4B). Further support for the premise that indeed Rli60 restricts BCAA biosynthesis was provided by the finding that the *rli60-ter* mutant grew better under low BCAA conditions, like *Δrli60* (*i*.*e*., better than WT bacteria), whereas the “super-attenuator” mutants grew similarly to *ΔilvC*, (*i*.*e*., worse than WT bacteria), in accordance with their predicted *ilv-leu* gene regulation (Fig 4 and S4 Fig). Taken together, these results demonstrated that *Lm* is capable of relying completely on *de novo* BCAA synthesis and grow independently of external BCAA, though this capability is restricted by Rli60.

### Restriction of BCAA biosynthesis promotes *Lm* virulence

**Fig 5:**
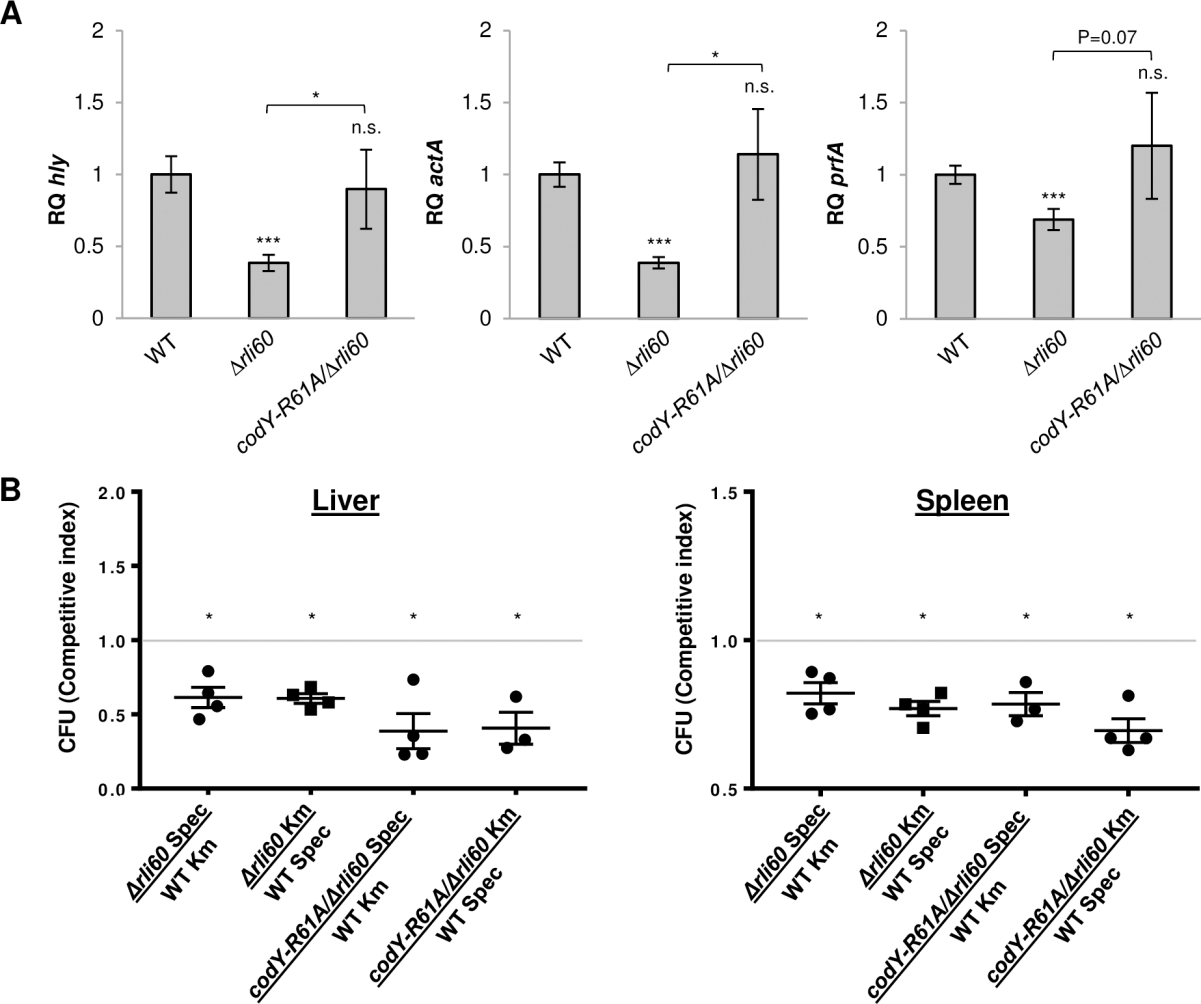
Restriction of BCAA biosynthesis promotes *Lm* virulence. **(A)** qRT-PCR analysis of *hly*, *actA* and *prfA* transcription levels in WT *Lm* and indicated mutants grown in LBMM. mRNA levels are represented as relative quantity (RQ) and normalized to *rpoD*. The data represent 4 biological replicates (N = 4). Error bars indicate standard deviation. Asterisks represent *P*-values (* = P<0.05, ** = P<0.01, *** = P<0.001, n.s. = non-significant), calculated using Student’s *t*-test. **(B)** *Δrli60* and *codY-R61A/Δrli60* bacteria are attenuated for virulence compared to WT *Lm* bacteria during *in-vivo* competitive infection of mice. Bacterial loads in the spleens and livers of C57BL/6 mice after 2 days of infection with a 1:1 ratio of WT *Lm* and mutant bacteria (either *Δrli60* or *codY-R61A/Δrli60*). pPL2 containing a Kanamycin (Km) or Spectinomycin (Spec) resistance cassette were used to identify each bacterial strain. The experiment was done reciprocally, switching the antibiotic cassete between the two strains. Colony forming units (CFU) of mutant bacteria in each organ are presented as relative to the CFU of WT bacteria and are significantly different from CFU (WT/Mutant) = 1, calculated using Student’s *t*-test (* = p<0.05). Mutant Km/WT Spec CFU is not significantly different from the reciprocal Mutant Spec/WT Km CFU.

To investigate whether BCAA semi-auxotrophy supports *Lm* virulence, we next analyzed the transcription of three major virulence genes, *prfA*, *hly* and *actA*, in WT and *Δrli60* bacteria grown in LBMM (that mimicks intracellular conditions [19]). Notably, the transcription level of the virulence genes was reduced in *Δrli60* in comparison to WT bacteria (Fig 5A), suggesting that over-production of BCAA hinders virulence gene expression. Of note, in a previous study that examined the impact of Rli60 on virulence gene expression, an enhanced *prfA* transcription was detected in a *Δrli60* mutant [29]. A close examination of this *rli60* deletion mutant indicated that the *ilvD* TSS was also deleted together with the *rli60* sequence (*ilvD* TSS was identified here by 5'-RACE analysis, S2 Fig), therefore it is most likely that BCAA biosynthesis was impaired in this resulting mutant, which can indeed further lead to enhanced *prfA* transcription by CodY.

To examine whether the reduction in transcription of virulence genes in response to Rli60 deletion is mediated by CodY, we combined *Δrli60* deletion with R61A mutation in CodY, which considerably reduces CodY’s affinity to isoleucine (*codY-R61A/Δrli60* mutant) [20,34], and tested this double mutant for virulence gene expression. We reasoned that the mutated CodY, will be “blind” to the increase in isoleucine and therefore, virulence genes will be activated. In line with our prediction, we found the double mutant to induce virulence gene transcription similarly to WT bacteria (Fig 5A), supporting the premise that uncontrolled production of BCAA directly affects CodY regulation, hindering its ability to activate virulence gene expression under low BCAA conditions. Unlike the experiments in defined medium, examination of *Δrli60* and *codY-R61A/Δrli60* mutants *in vivo* in mice infections implied a more complex picture. Both mutants were slightly attenuated for virulence in comparison to WT bacteria, demonstrating ~40% reduction in competitive fitness, as evaluated using a competitive index assay (Fig 5B). While we previously found that *ΔcodY* is 10-fold less virulent in mice (whereas *prfA* mutant is >100-fold less virulent) [20,35], it is likely that the *codY-R61A* and *rli60* mutations only partially alter CodY activity and thus lead to a slight effect *in vivo*. We previously demonstrated that CodY functions both in its isoleucine-bound and -unbound form, simultaneously activating and repressing different genes, some of which are important for virulence independently of PrfA, therefore affecting *Lm* gene expression in a highly complex manner [23]. Moreover, within the intracellular niche BCAA are not the sole signal for *prfA* activation, and multiple signals were shown to play a role, which together orchestrate virulence gene expression. This study focuses on one such signal, overall demonstrating that BCAA biosynthesis fine tunes CodY activity and thereby virulence gene transcription.

## Discussion

Bacteria rarely encounter rich nutrient conditions in natural environments. Bacterial pathogens that traverse freely between extracellular and intracellular environments are frequently subjected to massive changes in nutrient availability. The ability to sense nutrients, remodel metabolic pathways and change behavior via gene regulation is therefore fundamental to bacterial adaptation and growth. Furthermore, nutrient sensing provides essential information regarding the physiological condition of the environment, signaling a niche specific “signature” that informs the bacteria of their exact location (*e*.*g*., extracellular *vs*. intracellular). This added information is particularly critical during host invasion, as pathogens need to quickly express virulence factors to counteract host defense mechanisms in order to survive. In line with this premise, this study demonstrates that sensing of BCAA is an important feature of *Lm* not only to support growth but also to promote virulence, and that the ability to control BCAA production is fundamental to successful invasion. It is generally accepted that controlled metabolite production is crucial for cell functioning and growth by providing competitive advantage in natural environments. Yet, here we show that *Lm* has evolved a regulatory mechanism for BCAA biosynthesis that hampers growth in extracellular environments but gives an advantage within the host. Limiting *de novo* BCAA biosynthesis enables CodY to accurately sense the external level of isoleucine and to regulate genes in a BCAA-concentration dependent manner. In a sense, this tightly regulated BCAA auxotrophy of *Lm* has become a control point that shapes not only metabolic networks but also virulence gene expression and thus the ability of *Lm* to infect its host. We propose that this adaptive mechanism may be the result of co-evolution of *Lm* with its host, allowing isoleucine to be used as a host specific signal.

**Fig 6:**
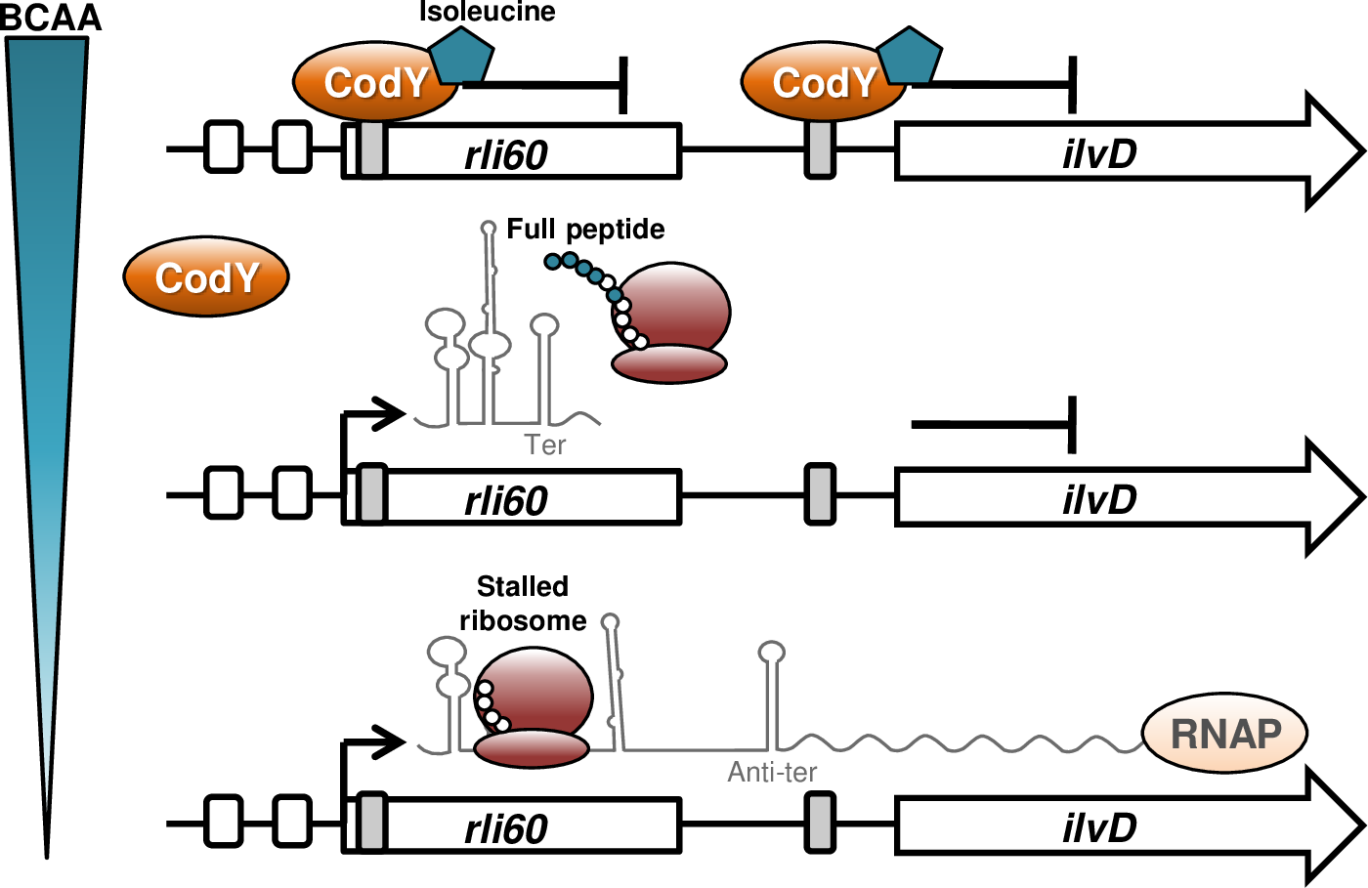
Sequential regulation of the *ilv-leu* operon. A schematic model of the *ilv-leu* operon regulation. In *Lm* the BCAA biosynthesis pathway is regulated by two sequential mechanisms, the first using the classical CodY repression under rich BCAA conditions and the second using Rli60-ribosome-mediated attenuation under poor BCAA conditions. Upon a drop in BCAA levels, CodY repression is alleviated, *rli60* is transcribed, forming two alternative RNA structures that terminate or anti-terminate the transcription of the downstream genes. Transcription attenuation is dictated by the leader peptide translating ribosome as classically shown for translation-coupled ribosome-mediated attenuation. This mechanism relies on two types of regulators, a global *i*.*e*., CodY and a specific *i*.*e*., Rli60. RNAP, RNA polymerase.

The finding that isoleucine deficiency is the signal for virulence gene activation, prompted us to look for mechanisms that control isoleucine biosynthesis during infection. We knew that BCAA biosynthesis in *Lm* is intact and functional and that the *ilv-leu* genes are up-regulated when BCAA levels drop [19,25]. However, while CodY was shown to regulate the *ilv-leu* genes under rich nutrient conditions, it was not clear if and how BCAA biosynthesis is controlled under poor nutrient conditions, *e*.*g*. within the host. In this study, we characterized Rli60 as a ribosome-mediated attenuator that controls the *ilv-leu* gene transcription in a BCAA-dependent manner. While many bacteria use attenuation mechanisms as ON/OFF switches to regulate amino acid biosynthesis [31,33,36], we found Rli60 to limit BCAA production such that internal levels are insufficient to support optimal growth. This BCAA auxotrophy of *Lm* is partial, fully dependent on Rli60, thus falling into the category of 'phenotypic auxotrophy', whereby auxotrophy is a result of gene dysregulation rather than loss of function [37]. Overall, our findings indicate that BCAA biosynthesis in *Lm* is regulated by two mechanisms, the first involving classical CodY repression under nutrient rich conditions and the second using Rli60-ribosome-mediated attenuation under poor BCAA conditions (Fig 6). This model relies on two types of regulations; a global (via CodY) and a specific (via Rli60), which is typical for metabolic pathways. However, since isoleucine (the end product of this pathway) is also the input signal of CodY, Rli60 has the capacity to impact CodY activity, and thereby global gene expression, strengthening the premise that BCAA production must be tightly regulated. In support of this idea, a previous study in *B*. *subtillis* has demonstrated that changes in endogenous BCAA biosynthesis indeed affect CodY global regulation [38].

Regulation of bacterial and host behaviors via amino acid auxotrophy is an emerging concept. For example, Group A *Streptococcus* bacteria (GAS) requires supplementation of asparagine to support growth and depends on the host supply [39]. Notably, it was shown that GAS stimulates host asparagine synthesis via secretion of hemolysin toxins that trigger endoplasmic reticulum stress. In parallel, GAS senses host derived asparagine, using a two-component system, and regulates metabolic and virulence genes, including the hemolysin toxin genes [39]. *Francisella tularensis* and *Legionella pneumophila* are additional example, as these bacteria have lost their ability to synthesize certain amino acids, but developed unique mechanisms to obtain them from the host [40]. *F*. *tularensis*, auxotrophic for BCAA, triggers the host macroautophagy degradation machinery to increase the intracellular pool of these amino acids [41]. Similarly, *L*. *pneumophila*, auxotrophic for seven amino acids (Arg, Cys, Ile, Leu, Met, Thr and Val) [42,43], triggers proteasomal degradation of polyubiquitinated proteins and activate mammalian transporters to import the required amino acids into the *Legionella* containing vacuole [44–46]. Although it is still not clear how *Legionella* and *Francisella* sense the availability of host nutrients and whether they use this information to regulate virulence, it is likely that such a mechanism exists. Of note, it was previously suggested that threonine signals *Legionella* to differentiate and replicate within macrophage cells [47]. Together these examples demonstrate how amino acid auxotrophy in bacterial pathogens can be a driving force of pathogenic evolution or an adaptive mechanism to life within the host, supporting the premise that metabolism and virulence are tightly interlinked.

Interestingly, the idea of amino acid auxotrophy as a system that regulates cellular responses exists also in mammalian cells. Humans and most mammals are auxotrophic for certain amino acids and acquire them from the microbiota and diet. It is clear now that this amino acid auxotrophy, particularly of immune cells, is involved in regulation of immune responses, production of antimicrobial effectors, T cell responses and additional mechanisms [48]. Several amino acids were shown to function as immuno-modulators such as arginine, tryptophan and glutamine. Arginine plays a role in macrophage activation and blocks tumor growth mainly via its conversion to nitric oxide (NO), which by itself is toxic to microbes and targets intracellular pathogens in addition to its signaling roles [49–51]. Tryptophan is degraded to kynurenines that were suggested to regulate T cells and glutamine was shown to be important for T cells proliferation [52–54]. In light of these findings, it could be interesting to examine how bacterial pathogens compete for these amino acids within the host taking into account their regulatory roles, potentially manipulating them to subvert host responses.

In conclusion, controlled BCAA auxotrophy in *Lm* likely represents an adaptive mechanism to the life within the host. This study places Rli60 at the cross-road of metabolism and virulence and validates the role of BCAA in *Lm* regulation of virulence. A better understanding of bacterial pathogens metabolism during infection and its links to virulence and host cell modulation is critical for our understanding of bacterial pathogenesis and for the identification of new metabolic targets that can be the basis for the development of novel drugs and therapeutic approaches.

## Materials and methods

### Ethics statement

Experimental protocols were approved by the Tel Aviv University Animal Care and Use Committee (01-15-052, 04-13-039) according to the Israel Welfare Law (1994) and the National Research Council guide (Guide for the Care and Use of Laboratory Animals 2010).

### Bacterial strains, plasmids and primers

*Listeria monocytogenes* 10403S was used as the WT strain and as the parental strain to generate allelic exchange mutant strains (S1 Table). *E*. *coli* XL-1 Blue strain (Stratagene) was used for generation of vectors, and *E*. *coli* SM-10 strain [55] was used for plasmid conjugation to *Lm*. Plasmids and primers used in this study are listed in S1 and S2 Tables, respectively.

### Growth conditions

*Lm* bacteria were grown at 37°C with agitation in brain heart infusion (BHI) as a rich medium or in minimal defined medium (MM), and harvested at mid logarithmic growth (OD_600_ of ~0.3). MM was prepared as described previously [56]: phosphate buffer (48.2 mM KH_2_PO_4_ and 153 mM Na_2_HPO_4_, pH 7), 0.41 mg/ml magnesium sulfate, 10 mg/ml glucose, 100 μg/ml of each amino acid (methionine, arginine, histidine, tryptophan, phenylalanine, cysteine, isoleucine, leucine and valine), 600 μg/ml glutamine, 0.5 mg/ml biotin, 0.5 mg/ml riboflavin, 20 mg/ml ferric citrate, 1 mg/ml para-aminobenzoic acid, 5 ng/ml lipoic acid, 2.5 mg/ml adenine, 1 mg/ml thiamine, 1 mg/ml pyridoxal, 1 mg/ml calcium pantothenate and 1 mg/ml nicotinamine. For growth under limiting concentrations of branched-chain amino acids (BCAA), MM was freshly made with 10-fold less of isoleucine, leucine and valine (resulting in a final concentration of 10 μg/ml or 80 μM of each amino acid) and named low-BCAA minimal defined medium (LBMM). For growth under limiting concentrations of either arginine or both phenylalanine and tryptophan, MM was freshly made with 10-fold less of these amino acids (resulting in a final concentration of 10 μg/ml). For growth curves, bacteria from overnight MM cultures were washed 3 times with PBS to remove excess BCAA and adjusted to OD_600_ of 0.03 in fresh MM without BCAA or supplemented with 2.5, 10, or 100 μg/ml of BCAA (20, 80 and 800 μM, respectively). Bacterial growth was measured by Synergy HT BioTek plate reader at 37°C for 55 h. OD_600_ measurements were taken every 15 min after shaking for 2 min.

### RNA extraction

Total RNA was extracted from bacteria using the RNA*snap* method [57]. Briefly, bacterial pellets were washed with AE Buffer (50 mM NaOAc pH 5.2, 10 mM EDTA) and then resuspended in 95% formamide, 18 mM EDTA, 1% 2-mercaptoethanol and 0.025% SDS. Bacterial lysis was performed by vortexing with 100 μm of zirconia beads (OPS Diagnostics) followed by incubation at 95°C. Nucleic acids were precipitated with ethanol and treated with Turbo-DNase (Ambion), followed by standard phenol extraction.

### Northern blot

PCR products of 152 and 970 bp for *rli60* and *ilvD*, respectively, were amplified from *Lm* genomic DNA using gene-specific primers for *rli60* and *ilvD* (S2 Table). Thirty nano-grams of each PCR product was used as a template for synthesis of ^32^P-labeled probes using NEblot kit (New England Biolabs) and ɑ-^32^P dCTP (PerkinElmer), according to manufacturer’s instructions. Equal amounts of total RNA (5–10 μg) were separated on 1% agarose gel containing 7.4% formaldehyde and stained with ethidium bromide for visualization of rRNA. RNA was transferred to Biodyne B 0.45 μM nylon membrane (Pall Life Sciences) and cross-linked by UV (0.12 Joules). Pre-hybridization was performed at 65°C for 2 h in Church buffer (sodium phosphate buffer 0.25 M pH = 7.2, 1% BSA, 1 mM EDTA, and 7% SDS). Probes were added to Church buffer and hybridization was performed overnight at 65°C. Membranes were washed with 2XSSC 0.1% SDS, 1XSSC 0.1% SDS and 1XSSC. Light sensitive films (Fuji) were exposed to radioactive membranes for visualization of RNA-probe hybridizations. Sizes of RNA bands were evaluated using Transcript RNA Markers 0.2–10 kb (Sigma-Aldrich).

### Quantitative RT-PCR

One μg of total RNA was reverse transcribed to cDNA using qScript (Quanta). qRT-PCR was performed on 10 ng of cDNA using FastStart Universal SYBR Green Master (Roche) in a StepOne Plus real time PCR system (Applied Biosystems). The transcription level of each gene was normalized to that of the reference gene *rpoD*. For the comparative analysis of *rli60* and *ilvD* transcripts, a standard curve was prepared using *Lm* genomic DNA.

### Rapid amplification of cDNA 5'-ends analysis (5'-RACE)

5'-RACE analysis was performed on total RNA extracts from *Lm* bacteria as described previously [58]. Briefly, 6 μg of total RNA were treated with Tobacco acid pyrophosphatase (TAP, Epicentre) and then ligated to a RNA linker using T4 RNA ligase 1 (Epicentre). TAP-untreated samples were analyzed in parallel in order to identify processed transcripts. Two μg of linker-ligated RNA were used for first-strand cDNA synthesis with random hexamers (Invitrogen) and Superscript III reverse transcriptase (Invitrogen). PCR amplification of the first-strand cDNA products was performed using a gene-specific primer (either *rli60* or *ilvD*) and a linker specific primer. PCR products were then separated on 3% agarose gels, and TAP-specific bands were purified and cloned into pUC-18 for sequence analysis.

### Transcription levels in intracellular bacteria

RNA was purified from intracellularly grown bacteria in bone marrow-derived macrophage cells (BMDM) as described previously [59]. BMDM cells used for infection experiments were isolated from 6–8 week-old female C57BL/6 mice (Envigo, Israel) as described previously [60] and cultured in Dulbecco’s Modified Eagle Medium (DMEM)-based media supplemented with 20% fetal bovine serum, sodium pyruvate (1 mM), L-glutamine (2 mM), β- Mercaptoethanol (0.05 mM) and monocyte-colony stimulating factor (M-CSF, L929-conditioned medium. Briefly, WT *Lm* bacteria were used to infect BMDM seeded in a 145 mm dish, resulting in a MOI of ~100. Thirty minutes after infection, BMDM monolayers were washed twice with PBS to remove unattached bacteria and fresh medium was added. At 1 h post-infection, gentamicin (50 μg/ml) was added to limit bacterial extracellular growth. 2 hours post infection, intracellular bacteria were collected using 0.45 μM filter membranes and flash-freezed in liquid nitrogen. Bacteria were recovered from filters by vortexing into AE buffer (50 mM NaOAc pH 5.2, 10 mM EDTA), and bacterial nucleic acids were extracted using hot (65°C) phenol with 1% SDS followed by ethanol precipitation. Rneasy Mini Kit Dnase on column (Qiagen) was used for Dnase treatment. Transcription levels of *rli60* and *ilvD* in total RNA samples were measured with specific probes using the NanoString nCounter system, according to manufacturer standard procedures [61]. Total RNA extracted from bacteria grown in BHI was analyzed in parallel as a control.

### Western blot analysis

WT *Lm* or indicated mutants (*ΔcodY*, *Δrli60* or *ΔcodY/Δrli60*) harboring 6his-tagged *ilvD* at its native locus (*ilvD*-6his) or the translational fusion of the leader peptide to enhanced green florescent protein (EGFP) on the integrative pPL2 plasmid (pPL2 *rli60-peptide-EGFP*) were grown as indicated. Cultures were washed with Buffer-A (20mM Tris-HCl pH = 8, 0.5M NaCl, and 1 mM EDTA), resuspended in 20 ml of Buffer-A supplemented with 1 mM PMSF and lysed by an ultra-high-pressure homogenizer (Stansted Fluid Power) at 12000 psi. Lysates were centrifuged at 3,000 rpm for 10 min at 4°C. Proteins from the supernatants were precipitated on ice for 1 hour using 10% TCA and centrifuged at 3,800 rpm for 30 min at 4°C. Supernatants were discarded and the pellets were washed in Buffer-A with 5% TCA, then washed with ice-cold acetone twice. Dried pellets were resuspended in water with 2% SDS and analyzed for total protein content by modified Lowry assay. Samples with equal amounts of total proteins were separated on 15% SDS-polyacrylamide gels and transferred to nitrocellulose membranes. Proteins were probed either with mouse anti-6His tag (Abcam ab18184) or anti-GFP (Covance, a kind gift from E. Bacharach lab, Tel Aviv University) antibody used at a 1:1000 dilution, followed by HRP-conjugated goat anti-mouse IgG (Jackson ImmunoResearch, USA) at a 1:20,000 dilution. Homemade anti-GroEL antibody (a kind gift from A. Azem lab, Tel Aviv University) was used as an internal control at a dilution of 1:20,000, followed by HRP-conjugated goat anti-rabbit IgG at a dilution of 1:20,000. Western blots were developed by enhanced chemiluminescence reaction (ECL). ImageJ software (https://imagej.nih.gov/ij/) was used for densitometry of obtained bands.

### Lux reporter assay

Overnight *E*. *coli* K-12 *ilvC*::*Km* bacteria (Keio collection, a kind gift from U. Qimron lab, Tel Aviv University) harboring the *rli60*-luciferase reporter system (pPL2*-rli60-luxABCDE* or pPL2*-Δrli60-luxABCDE*) grown in MM cultures were adjusted to OD_600_ of 0.03 in fresh MM medium supplemented with 1, 10, or 100 μg/ml of BCAA (8, 80 and 800 μM, respectively), and grown in a Synergy HT BioTek plate reader at 37°C for 12 h. Luminescence measurements at 12 h time point at the different BCAA concentrations were normalized to the corresponding OD_600_.

### DNA and RNA sequence analyses

*Lm ilvD* promoter was predicted using BPROM [62]. The leader peptide was predicted using ApE (http://biologylabs.utah.edu/jorgensen/wayned/ape). Terminator and anti-terminator structures were predicted using PASIFIC [32], with the kind help of Adi Millman from the Rotem Sorek lab, Weizmann institute. A scheme of the structures was prepared using Mfold [63].

### *In vivo* competitive index experiments in mice

Competitive index assay was performed as described previously [64]. Briefly, WT *Lm*, *Δrli60* and *codY-R61A/Δrli60* bacteria harboring the integrative pPL2 plasmid containing a kanamycin or spectinomycin resistance genes were grown in BHI medium at 30°C overnight. Bacterial cultures were washed in PBS, measured for OD_600_ and mutant culture (either *Δrli60* or *codY-R61A/Δrli60*) was mixed with WT culture at a 1:1 ratio. Eight weeks old C57BL/6 female mice (Envigo) were infected via tail vein injections with 4 × 10^4^ total bacteria in 200 μl of PBS. Animals were observed daily for any signs of illnesses and were euthanized 2 days post-infection. Spleens and livers were harvested and homogenized in 0.2% Triton X-100 in PBS, and the numbers of viable bacteria in each organ were determined by plating serial dilutions of homogenates onto BHI agar plates containing kanamycin or spectinomycin. The experiment was performed twice using five mice in each group per experiment.

## Supporting information

S1 Fig The leader sequence of the *ilv-leu* operon. The regulatory region upstream to *ilvD* gene is shown. The following elements are marked: the -35 and -10 promoter sites, *rli60* sequence, CodY-binding sites, the putative terminator structure and *ilvD* RBS and ATG are highlighted. Also indicated are the transcription start site (TSS), as determined by the 5’-RACE (see S2 Fig), the leader peptide ribosome binding site (RBS), start codon (ATG), stop codon (TAG, indicated by an asterisk), and a putative terminator.
(TIF)

S2 Fig 5‘-RACE analysis of the *ilv-leu* promoter region. **(A)** 5'-RACE assay in WT bacteria grown in BHI, MM and LBMM. Schematic representation of the location of *rli60*- and *ilvD*-specific primers (rli60_5'RACE and ilvD_5'RACE respectively, see S2 Table) used for amplification of the transcription start site (TSS) (Upper panel). 5’-RACE PCR products, obtained with a linker- specific primer (linkerS_5'RACE, see S2 Table) and either *rli60*- or *ilvD*- specific primer, separated on a 3% agarose gel (lower panel). TAP, tobacco acid pyrophosphatase. Sizes of TAP-specific products that represent primary (unprocessed) transcripts are indicated with arrows. An asterisk indicates a product with the same TSS as the 500 bp product. **(B)** Schematic representation of the *rli60* and *ilvD* transcripts in bacteria grown in MM and BHI media, with their transcription start site, based on the sequence of 5’-RACE products.
(TIF)

S3 Fig *ilvD* is specifically upregulated upon BCAA limiting conditions. qRT-PCR analysis of *ilvD* transcription in WT *Lm* grown in MM, LBMM and MM medium containing low levels of arginine or both phenylalanine and tryptophan. mRNA levels are represented as relative quantity (RQ), relative to *ilvD* mRNA level in WT bacteria grown in MM. *ilvD* mRNA levels were normalized to *rpoD* mRNA. The data represent 3 biological replicates (N = 3). Error bars indicate standard deviation. Asterisks represent *P*-values (* = P<0.05, ** = P<0.01, *** = P<0.001, n.s. = non-significant), calculated using Student’s *t*-test. *P*-values represent a comparison to the WT MM sample.
(TIF)

S4 Fig Bacterial growth curves in minimal defined medium supplemented with varying BCAA concentrations. Growth of WT *Lm* and indicated mutants under decreasing concentration of BCAA (800 μM, 80 μM, 20 μM) in a minimal defined medium, as measured by Synergy HT BioTek plate reader at 37°C for 55 h. Bacterial cultuers were pre-grown over night in MM medium, washed extensively and diluted to OD_600_ of 0.03 for growth. The data represent 3 biological replicates (N = 3). Error bars represent standard deviation.
(TIF)

S1 Table Strains and plasmids used in this study. (PDF)

S2 Table Oligonucleotides used in this study. (PDF)
