# Supplementary figures and images for: Controlled branched-chain amino acids auxotrophy in Listeria monocytogenes allows isoleucine to serve as a host signal and virulence effector
Source: PLoS Genet. 2018 Mar 12;14(3):e1007283. doi: 10.1371/journal.pgen.1007283 (PMC5864092; doi:10.1371/journal.pgen.1007283)

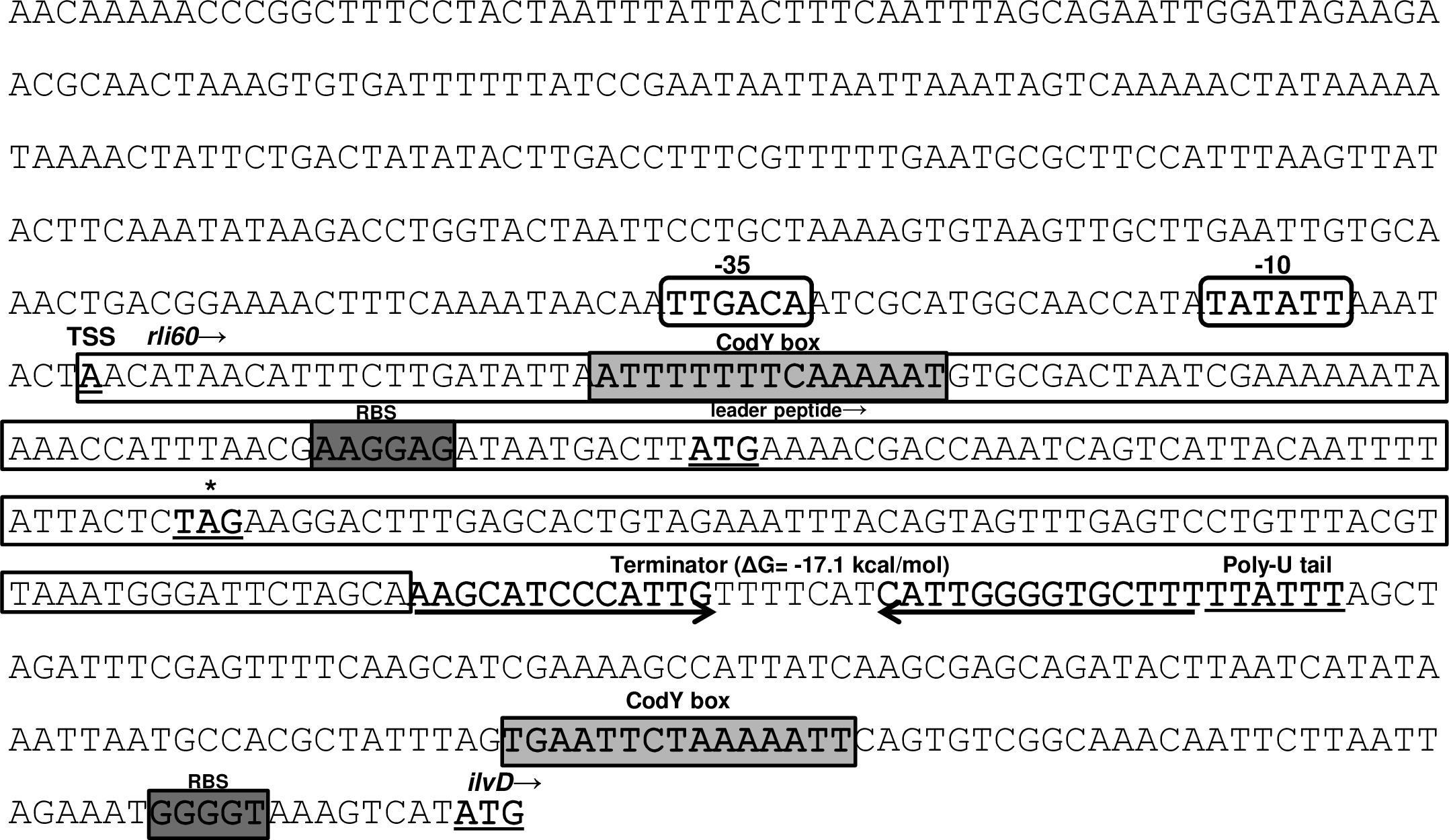

Supplement: S1 Fig — The regulatory region upstream to ilvD gene is shown. The following elements are marked: the -35 and -10 promoter sites, rli60 sequence, CodY-binding sites, the putative terminator structure and ilvD RBS and ATG are highlighted. Also indicated are the transcription start site (TSS), as determined by the 5’-RACE (see S2 Fig), the leader peptide ribosome binding site (RBS), start codon (ATG), stop codon (TAG, indicated by an asterisk), and a putative terminator. (TIF) [file pgen.1007283.s001.tif]

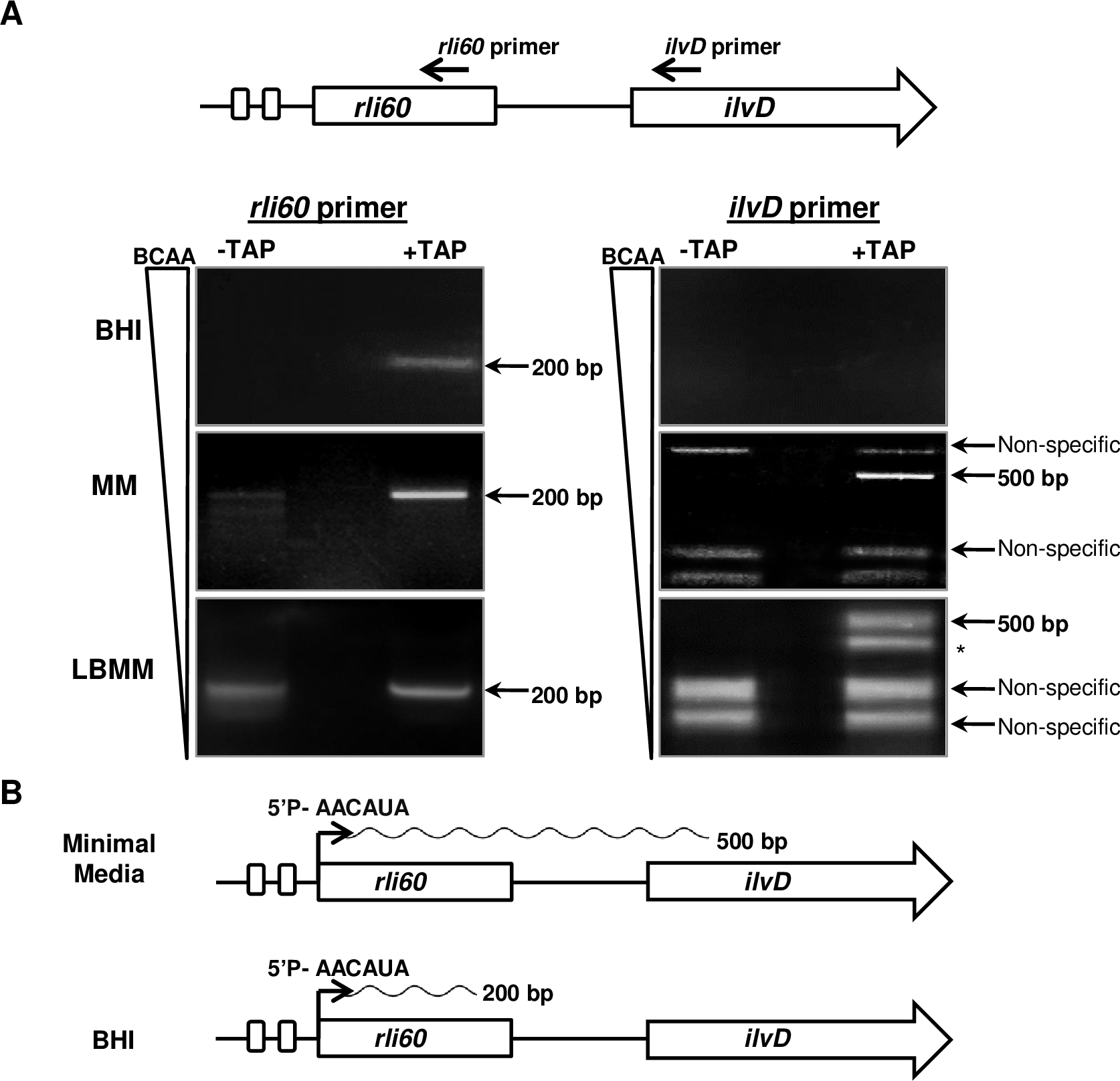

Supplement: S2 Fig — (A) 5'-RACE assay in WT bacteria grown in BHI, MM and LBMM. Schematic representation of the location of rli60- and ilvD-specific primers (rli60_5'RACE and ilvD_5'RACE respectively, see S2 Table) used for amplification of the transcription start site (TSS) (Upper panel). 5’-RACE PCR products, obtained with a linker- specific primer (linkerS_5'RACE, see S2 Table) and either rli60- or ilvD- specific primer, separated on a 3% agarose gel (lower panel). TAP, tobacco acid pyrophosphatase. Sizes of TAP-specific products that represent primary (unprocessed) transcripts are indicated with arrows. An asterisk indicates a product with the same TSS as the 500 bp product. (B) Schematic representation of the rli60 and ilvD transcripts in bacteria grown in MM and BHI media, with their transcription start site, based on the sequence of 5’-RACE products. (TIF) [file pgen.1007283.s002.tif]

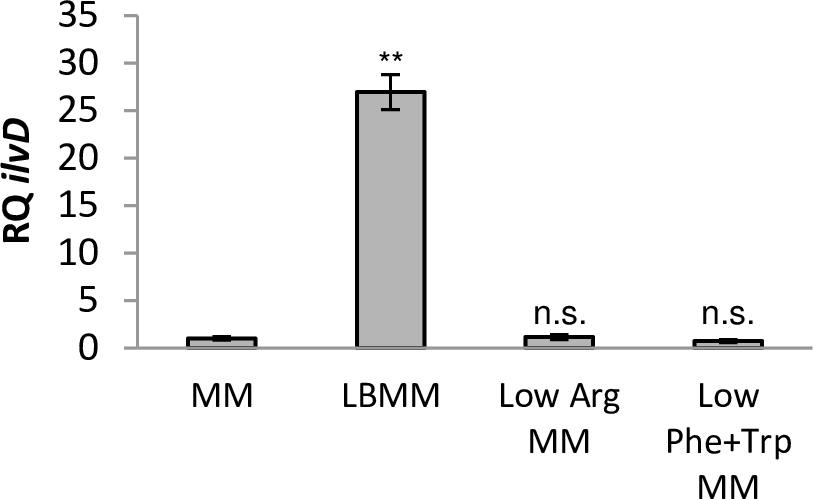

Supplement: S3 Fig — qRT-PCR analysis of ilvD transcription in WT Lm grown in MM, LBMM and MM medium containing low levels of arginine or both phenylalanine and tryptophan. mRNA levels are represented as relative quantity (RQ), relative to ilvD mRNA level in WT bacteria grown in MM. ilvD mRNA levels were normalized to rpoD mRNA. The data represent 3 biological replicates (N = 3). Error bars indicate standard deviation. Asterisks represent P-values (* = P<0.05, ** = P<0.01, *** = P<0.001, n.s. = non-significant), calculated using Student’s t-test. P-values represent a comparison to the WT MM sample. (TIF) [file pgen.1007283.s003.tif]

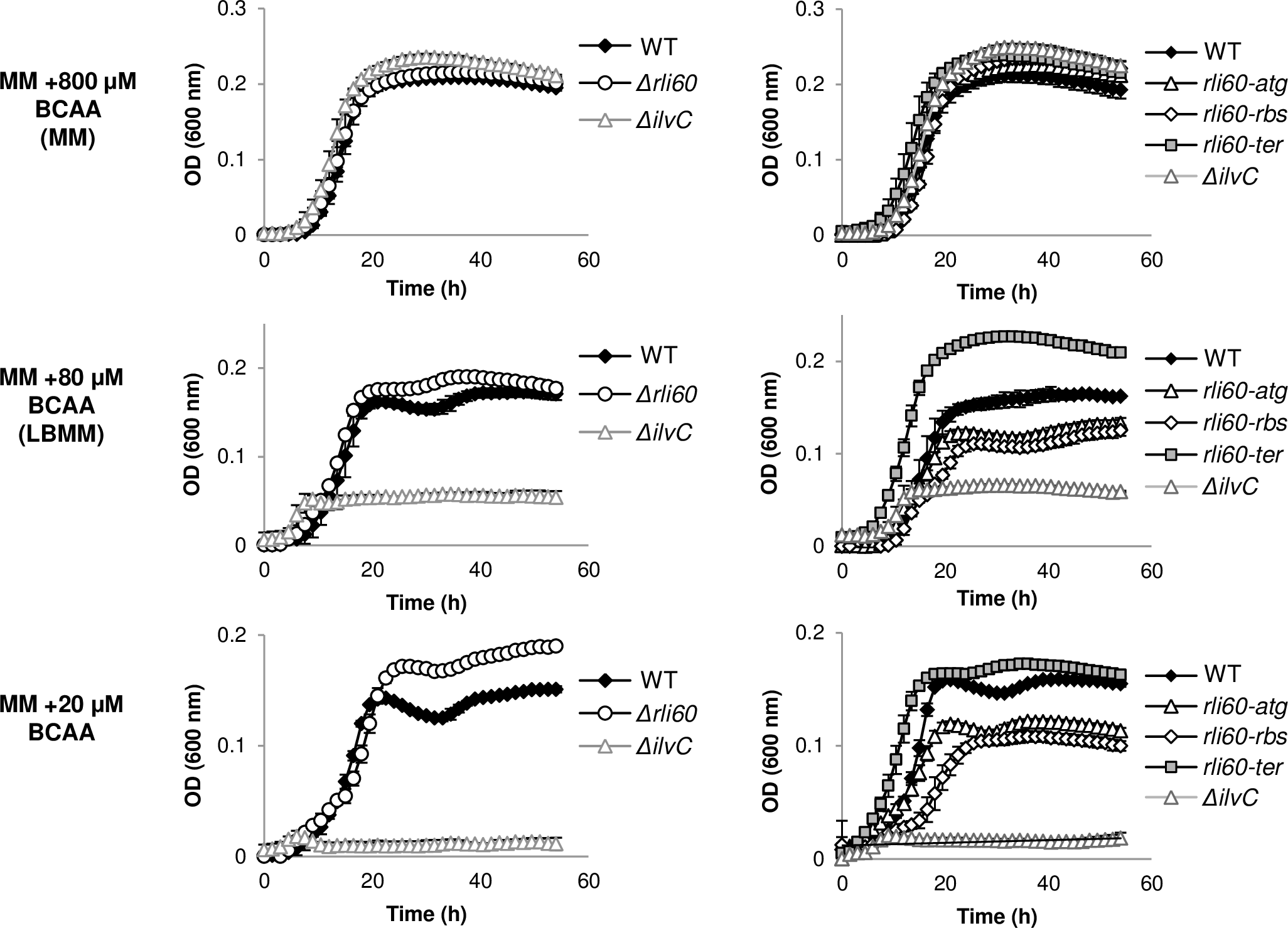

Supplement: S4 Fig — Growth of WT Lm and indicated mutants under decreasing concentration of BCAA (800 μM, 80 μM, 20 μM) in a minimal defined medium, as measured by Synergy HT BioTek plate reader at 37°C for 55 h. Bacterial cultuers were pre-grown over night in MM medium, washed extensively and diluted to OD600 of 0.03 for growth. The data represent 3 biological replicates (N = 3). Error bars represent standard deviation. (TIF) [file pgen.1007283.s004.tif]
