# Supplementary material for: Controlled branched-chain amino acids auxotrophy in Listeria monocytogenes allows isoleucine to serve as a host signal and virulence effector
Source: PLoS Genet. 2018 Mar 12;14(3):e1007283. doi: 10.1371/journal.pgen.1007283 (PMC5864092; doi:10.1371/journal.pgen.1007283)
Supplement: S1 Table — (PDF) [file pgen.1007283.s005.pdf]

S1 Table. Strains and plasmids used in this study

**Strain**	**Description**	**Source**
L. monocytogenes 10403S	WT *L. monocytogenes*	D. Portnoy lab
ΔcodY	*codY* (*LMRG_00730*) deletion strain	[19]
ΔilvC	*ilvC* (*LMRG_01134*) deletion strain, a BCAA auxotroph	[19]
Δrli60	*rli60* complete deletion strain (220 nt deletion)	This study
ΔcodY/Δrli60	*codY* and *rli60* double deletion strain	This study
codY-R61A	Mutated in CodY BCAA-binding pocket, substitution of arginine-61 to alanine	[20]
codY-R61A/Δrli60	*codY-R61A* and *rli60* double mutant	This study
WT ilvD-6his	C terminus 6-his tagged *ilvD* (at the native locus)	This study
ΔcodY ilvD-6his	*codY* deletion strain with 6-his tagged *ilvD*	This study
Δrli60 ilvD-6his	*rli60* deletion strain with 6-his tagged *ilvD*	This study
ΔcodY/Δrli60 ilvD-6his	*codY* and *rli60* double deletion strain with 6-his tagged *ilvD*	This study
WT pPL2 rli60-peptide-EGFP	WT harboring pPL2 *rli60-peptide-EGFP*	This study
ΔcodY pPL2 rli60-peptide-EGFP	*codY* deletion strain harboring pPL2 *rli60-peptide-EGFP*	This study
rli60-ter	CAUUGGGGUGC→**G**AUU**C**G**C**GU**C**C substitution in the stem of the terminator (at the native locus)	This study
rli60-atg	AUG→A**A**C substitution in the start codon of the leader peptide (at the native locus)	This study
rli60-rbs	AAGGAGA→ AA**CC**A**C**A substitution in the ribosome binding site of the leader peptide (at the native locus)	This study
WT pPL2 Spec	WT harboring pPL2 Spec	This study
WT pPL2 Km	WT harboring pPL2 Km	This study
Δrli60 pPL2 Spec	*rli60* deletion strain harboring pPL2 Spec	This study
Δrli60 pPL2 Km	*rli60* deletion strain harboring pPL2 Km	This study
codY-R61A/Δrli60 pPL2 Spec	*codY-R61A* and *rli60* double mutant harboring pPL2 Spec	This study
codY-R61A/Δrli60 pPL2 Km	*codY-R61A* and *rli60* double mutant harboring pPL2 Km	This study

E. coli XL-1 Blue	Plasmid propagation strain	Stratagene
E. coli SM-10	For plasmid conjugation to *L. monocytogenes*	[55]
E. coli K-12 ilvC::Km	Mutated in *ilvC*, BCAA auxotroph strain	Keio collection (obtained from U. Qimron lab)
E. coli K-12 ilvC::Km pPL2 rli60-luxABCDE	Mutated in *ilvC*, a BCAA auxotroph strain harboring pPL2 *rli60-luxABCDE*	This study
E. coli K-12 ilvC::Km pPL2 Δrli60-luxABCDE	Mutated in *ilvC*, a BCAA auxotroph strain harboring pPL2 *Δrli60-luxABCDE*	This study

**Plasmid**	**Description**	**Source**
pBHE261 (pKS7 oriT)	A conjugative plasmid for delivery to *L. monocytogenes*, used for generating clean deletion/substitution strains	[55]
pUC18	A plasmid used for sequencing of 5'RACE products	Thermo Fisher Scientific
pPL2	A chromosomally integrative plasmid of *L. monocytogenes*	[65]
pPL2 *luxABCDE*	A promoterless Luciferase reporter system cloned in pPL2	[66]
pPL2 *rli60-luxABCDE*	*rli60* locus fused to a luciferase reporter system in pPL2	This study
pPL2 *Δrli60-luxABCDE*	*Δrli60* locus fused to a luciferase reporter system in pPL2	This study
pPL2 *EGFP*	An enhanced green fluorescence protein cloned in pPL2	This study
pPL2 *rli60-peptide-EGFP*	An in-frame fusion of *rli60* leader peptide to EGFP cloned in pPL2	This study
pPL2 Spec	A Spectinomycin resistance cassette cloned in pPL2, for mice infections	This study
pPL2 Km	A Kanamycin resistance cassette cloned in pPL2, for mice infections	This study

Additional Supporting Information References

65. Construction, characterization, and use of two *Listeria monocytogenes* site-specific pahge integration vectors. *J Bacteriol* 184(15):4177–4186.

66. Bron PA, Monk IR, Corr SC, Hill C, Gahan CGM (2006) Novel luciferase reporter system for in vitro and organ-specific monitoring of differential gene expression in *Listeria monocytogenes*. *Appl Environ Microbiol* 72(4):2876–2884.
