# Supplementary material for: Controlled branched-chain amino acids auxotrophy in Listeria monocytogenes allows isoleucine to serve as a host signal and virulence effector
Source: PLoS Genet. 2018 Mar 12;14(3):e1007283. doi: 10.1371/journal.pgen.1007283 (PMC5864092; doi:10.1371/journal.pgen.1007283)
Supplement: S2 Table — (PDF) [file pgen.1007283.s006.pdf]

S2 Table. Oligonucleotides used in this study

**Primer Name**	**Sequence**	**Description**
RNA linker_5'RACE	GACGAGCACGAGGACACTGACATGGAGCTCGGAGTAGAAA	RNA linker for 5'RACE
linkerS_5'RACE	AAAAGAGCTGACTGACATGGAGCTCGGA	Linker specific primer for 5'RACE + SacI site for cloning into pUC-18
rli60_5'RACE	AAAACTGCAGCCCATTTAACGTAAACAGGACTC	*rli60* specific primer for 5'RACE + PstI site for cloning into pUC-18
ilvD_5'RACE	AAAACTGCAGATGGTTTGTCCATATCACCTGG	*ilvD* specific primer for 5'RACE + PstI site for cloning into pUC-18
rli60_Northern_F	CGACTAATCGAAAAAATAAAACCATTTAACG	For Northern blot probe
rli60_Northern_R	GAATCCCATTTAACGTAAACAGGAC	For Northern blot probe
ilvD_Northern_F	AAGCGCTCACTCTTTCTTCTG	For Northern blot probe
ilvD_Northern_R	ATACCGCGAGTAGCTCCA	For Northern blot probe
ilvD_6his_A	ATATGGATCCAGATGCGATTGATGATGCTTTC	For *ilvD-6his* + BamHI site for cloning into pBHE261
ilvD_6his_B	GTGGTGGTGGTGGTGGTGCTCGAGGTCGATTAAATCTTCTGGAATTT	For IlvD-6His
ilvD_6his_C	CTCGAGCACCACCACCACCACCACTGAAAAATGAGGTGATAAGCGTG	For IlvD-6His
ilvD_6his_D	GCGCTTCACCTTGCGTAATTTC	For IlvD-6His
rli60_lux_A	ATATGGGCCCAACAAAAACCCGGCTTTCCTA	For *rli60-luxABCDE* + PspOMI site for cloning into pPL2

rli60_lux_D	TTTTGTCGACCCCATATGACTTTACCCCATTTC	For *rli60-luxABCDE* + SalI site for cloning into pPL2
Del_rli60_lux_B	CTTTTGAGCTTAATTATAGTTCTTTACAATACAATCATAAATTATATATACCAACG	For *Δrli60-luxABCDE*
Del_rli60_lux_C	TAACATTTCTTGATATTAATTCGAGTTTTCAAGCATCGAAAAGC	For *Δrli60-luxABCDE*
rli60_pep_EGFP_A	ATATGGGCCCAACAAAAACCCGGCTTTCCTA	For *rli60-peptide-EGFP* + PspOMI site for cloning into pPL2
rli60_pep_EGFP_B	AGTGAAAAGTTCTTCTCCTTTACTCCCCCCGAGTAATAAAATTGTAATGACTGATTTGGT	For *rli60-peptide-EGFP*
rli60_pep_EGFP_C	ACCAAATCAGTCATTACAATTTTATTACTCGGGGGGAGTAAAGGAGAAGAACTTTTCACT	For *rli60-peptide-EGFP*
rli60_pep_EGFP_D	ATATGTCGACTTATTTGTATAGTTCATCCATGCCA	For *rli60-peptide-EGFP* + SalI site for cloning into pPL2
rli60_A	GACATGATTACGAATTCGAGCTCGGATTCCCACATCATCACTCTTCCTTG	For *rli60* deletion + SacI site for cloning into pBHE261
rli60_B	CTTTTGAGCTTAATTATAGTTCTTTACAATACAATCATAAATTATATATACCAACG	For *rli60* deletion
rli60_C	TAACATTTCTTGATATTAATTCGAGTTTTCAAGCATCGAAAAGC	For *rli60* deletion
rli60_D	GAGATCTCCTAGGGGCCCATCGCAAGGTTTAGCATCAGAAGTTATG	For *rli60* deletion + XmaI site for cloning into pBHE261
rli60-ter_A	ATATGAGCTCCAAAAACCCGGCTTTCCTA	For *rli60-ter* substitution mutant + SacI site for cloning into pBHE261
rli60-ter_B	TAAAAAGGACGCGAATCATGAAAACAATGGGATGCTTTGCTAGA	For *rli60-ter* substitution mutant

rli60-ter_C	TCATGATTCGCGTCCTTTTTATTTAGCTAGATTTCGAGTTTTCA	For *rli60-ter* substitution mutant
rli60-ter_D	ATATCTGCAGTTGCTGGAGCTTGTTCGA	For *rli60-ter* substitution mutant + PstI site for cloning into pBHE261
rli60-atg_A	ATATGAGCTCCAAAAACCCGGCTTTCCTA	For *rli60-atg* substitution mutant + SacI site for cloning into pBHE261
rli60-atg_B	TGATTTGGTCGTTTTCAGAAGTCATTATCTCCTTCGTTAAATGG	For *rli60-atg* substitution mutant
rli60-atg_C	AGGAGATAATGACTTCTGAAAACGACCAAATCAGTCA	For *rli60-atg* substitution mutant
rli60-atg_D	ATATCTGCAGTTGCTGGAGCTTGTTCGA	For *rli60-atg* substitution mutant + PstI site for cloning into pBHE261
rli60-rbs_A	ATATGAGCTCCAAAAACCCGGCTTTCCTA	For *rli60-rbs* substitution mutant + SacI site for cloning into pBHE261
rli60-rbs_B	TAAGTCATTATGTGGTTCGTTAAATGGTTTTATTTTTTCG	For *rli60-rbs* substitution mutant
rli60-rbs_C	AACCATTTAACGAACCACATAATGACTTATG	For *rli60-rbs* substitution mutant
rli60-rbs_D	ATATCTGCAGTTGCTGGAGCTTGTTCGA	For *rli60-rbs* substitution mutant + PstI site for cloning into pBHE261

Primers for RT-PCR

**Primer Name**	**Sequence**
rpoD_F	GGGGCTAATGAAAGCCGTTG
rpoD_R	CGCTTGACGAATCCACCACG
rli60_F	TGACTTATGAAAACGACCAAATCA
rli60_R	TCCCATTTAACGTAAACAGGACTC
ilvD_F	GATGAGGCTGTTGAGGCAAT
ilvD_R	GATACCGCGAGTAGCTCCAG
hly_F	TAAAAACAATGTATTAGTATACCACGG
hly_R	GATTCACAACTTGAATGTCTGC
actA_F	CAGCTAATAACGCAAACGGAAA
actA_R	CCTGGTTCTTCCTTCGCTTTT
prfA_F	ACGGGAAGCTTGGCTCTATTT
prfA_R	TGCCATCAGGAGTTTCTTTACCA
